# Lipolysis and Sterol Stability and Bioaccessibility of Wholemeal Rye Bread Enriched with Plant Sterols Subjected to Adult and Elderly Digestion Conditions

**DOI:** 10.1021/acs.jafc.4c03104

**Published:** 2024-07-22

**Authors:** Virginia Blanco-Morales, Dario Mercatante, Nerea Faubel, Diego Miedes, Mara Mandrioli, Maria Teresa Rodriguez-Estrada, Guadalupe Garcia-Llatas

**Affiliations:** † Nutrition and Food Science Area, Faculty of Pharmacy and Food Sciences, University of Valencia, Avda. Vicente Andrés Estellés s/n, Burjassot, 46100 Valencia, Spain; ‡ Department of Agricultural and Food Sciences, Alma Mater Studiorum-Università di Bologna, Viale Fanin 40, Bologna 40127, Italy

**Keywords:** INFOGEST, lipolysis, plant sterols, plant sterol oxidation products, senior population, adult

## Abstract

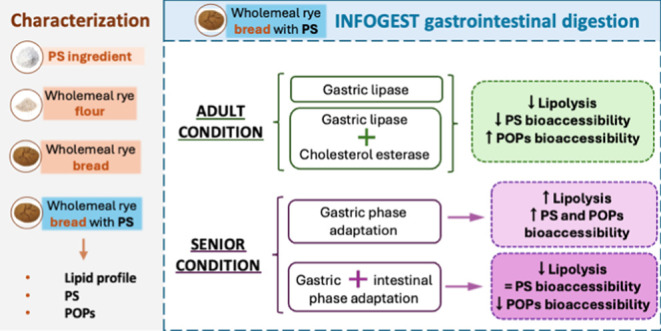

This study evaluated the impact of different digestion conditions (adult and senior) on lipolysis and bioaccessibility of plant sterols (PS) and phytosterol oxidation products (POPs) in PS-enriched wholemeal rye bread. Under adult digestion conditions, the addition of gastric lipase (GL) reduced lipolysis products (by 6.1% for free fatty acids and 11.7% for monoacylglycerols) and the bioaccessibility of PS by 6.7%, compared to the control. In digestion with both GL and cholesterol esterase (CE), these reductions were 12.9, 20.1, and 11.3%, respectively. Both modifications (GL and GL + CE) increased the bioaccessibility of POPs by 4.5–4.0%. When simulating the elderly digestion, the modified gastric and intestinal phases did not alter PS bioaccessibility but decreased POPs bioaccessibility by 21.8% compared to control, along with reduced lipolysis. Incorporating GL and CE thus approached physiological conditions and influenced lipid digestion. Elderly simulated digestion conditions resulted in a positive outcome by maintaining PS bioaccessibility while reducing potentially harmful POPs.

## 1 Introduction

Diet plays a pivotal role in human overall health, with a growing emphasis on promoting cardiovascular well-being through dietary choices.^1^ Among widely consumed cereals, rye (*Secale cereale* L.) grains have the highest fiber content, which is approximately 20% and is mainly composed of arabinoxylan, fructan, cellulose, and β-glucan.^2^ Due to its fiber content and composition, the European Food Safety Authority (EFSA) has authorized the following health claim for rye: “Rye fibre contributes to normal bowel function”.^3^ Rye is also an excellent source of essential nutrients and bioactive compounds, making wholemeal rye bread a dietary staple with numerous benefits, including improved digestion, weight management, and reduced risk of chronic diseases.^2,4^ The European Union has approved the market introduction of rye bread enriched with plant sterols (PS), bioactive compounds known for their cholesterol-lowering effects.^5,6^ The incorporation of PS into commonly consumed foods represents an interesting approach to potentially improve cardiovascular health.

Nevertheless, it is crucial to consider essential aspects of this combination, such as the PS susceptibility to oxidation, which may occur during food processing and storage. So far, no studies have assessed phytosterol oxidation product (POPs) formation during the preparation of PS-enriched wholemeal rye bread. In this regard, the baking process, which involves high temperatures, should be carefully considered due to its potential impact on POPs formation and, consequently, on the functionality of the final product.^7^ The bioactivity of PS-enriched foods is closely linked to their bioavailability, influenced by factors such as food matrix and digestion.^8^*In vitro* gastrointestinal digestion methods serve as predictive models to estimate the bioaccessibility of compounds like PS and POPs, crucial for optimizing their beneficial effects and minimizing potential harm.^9^

As the global aging population is rapidly increasing, it is essential to understand how physiological changes associated with aging affect nutrient digestion and absorption. For individuals over 65 years old, these changes can significantly influence their nutritional status and overall health.^10^ Therefore, developing food products tailored to the specific digestive capabilities and nutritional needs of the elderly is of great interest. Recent studies have shown that digestive efficiency diminishes with age due to factors like reduced enzyme activity and altered gastrointestinal conditions.^10,11^ Understanding how these changes affect the bioaccessibility of PS and POPs is crucial for optimizing dietary recommendations and improving health outcomes in the elderly.^11^

Recently, the bioaccessibility of PS after simulated gastrointestinal digestion under conditions mimicking the adult and senior population was evaluated using wholemeal rye bread as food matrix.^12,13^ Digestion under adult conditions was used to determine the effect of the addition of several key enzymes of lipid metabolism [gastric lipase (GL) and cholesterol esterase (CE)] on the bioaccessibility of PS. Furthermore, different digestion conditions (enzyme activity, pH levels, and bile concentration) can potentially affect the bioaccessibility of PS and POPs, as well as the release and absorption of lipolysis products such as monoacylglycerols (MAG) and free fatty acids (FFAs), among others.^14^ Thus, the determination of the major lipid classes after *in vitro* digestion is of great interest for understanding lipid fate in this process, which can affect the absorption of lipophilic bioactive compounds such as PS. However, to our knowledge, no studies have investigated the bioaccessibility of POPs and of the main lipid classes after the gastrointestinal digestion of PS-enriched food products, under conditions simulating either adults or the senior population.

Therefore, the present work aims to investigate how different digestion conditions, mimicking those of adults (with or without key lipid metabolism enzymes) and adapted to the senior population, impact the lipolysis process and the bioaccessibility of PS and POPs derived from PS-enriched rye bread.

## 2 Materials and Methods

### 2.1 Reagents

All solvents and reagents were of analytical grade and were purchased from Merck Life Science (Darmstadt, Germany); standards of triacylglycerols (TAG), diacylglycerols (DAG), monoacylglycerols (MAG), and enzymes were also supplied by Merck Life Science. Rabbit gastric extract was obtained from Lipolytech (Marseille, France). Fatty acid standard mix (GLC 412) was bought from Nu-Check Prep (Elysian, MN). PS standards were purchased from Merck Life Science and Avanti Polar Lipids (Alabaster, Alabama). Commercial standards of cholesterol oxidation products were supplied by Steraloids (Newport, RI).

### 2.2 Samples

A commercially available wholemeal rye flour (La Meta S.A., Barcelona, Spain) and two powdered ingredients from Lipofoods (Barcelona, Spain), one containing microencapsulated free PS and the other without PS (used as control), were utilized for the breadmaking procedure. Bread dough was prepared with whole rye flour, yeast, salt, water, and ascorbic acid, according to the proportions suggested by Makran et al.^15^ which included. The PS-enriched bread (PS-WRB) included 2.5% PS, while the control bread (WRB) had no PS enrichment. Both bread types were prepared according to a previously optimized procedure.^15^ Fresh bread samples were partially dehydrated and milled to achieve a more stable and disintegrated sample, thereby aiding in homogeneous sampling,^12^ and stored at −20 °C until further analysis.

### 2.3 Simulated Digestion

PS-WRB was subjected to different *in vitro* assays, including adult (18–65 years) and senior conditions (>65 years). Before conducting the digestion experiments, the enzyme activity and bile salts content required for preparing simulated fluids (salivary, gastric, and intestinal) were determined according to Minekus et al. protocol.^16^ The results of these determinations, along with the detailed digestion protocol, have been previously published.^12,17^ The digestion conditions assayed in this study are summarized in Table 1.

**Table 1:**
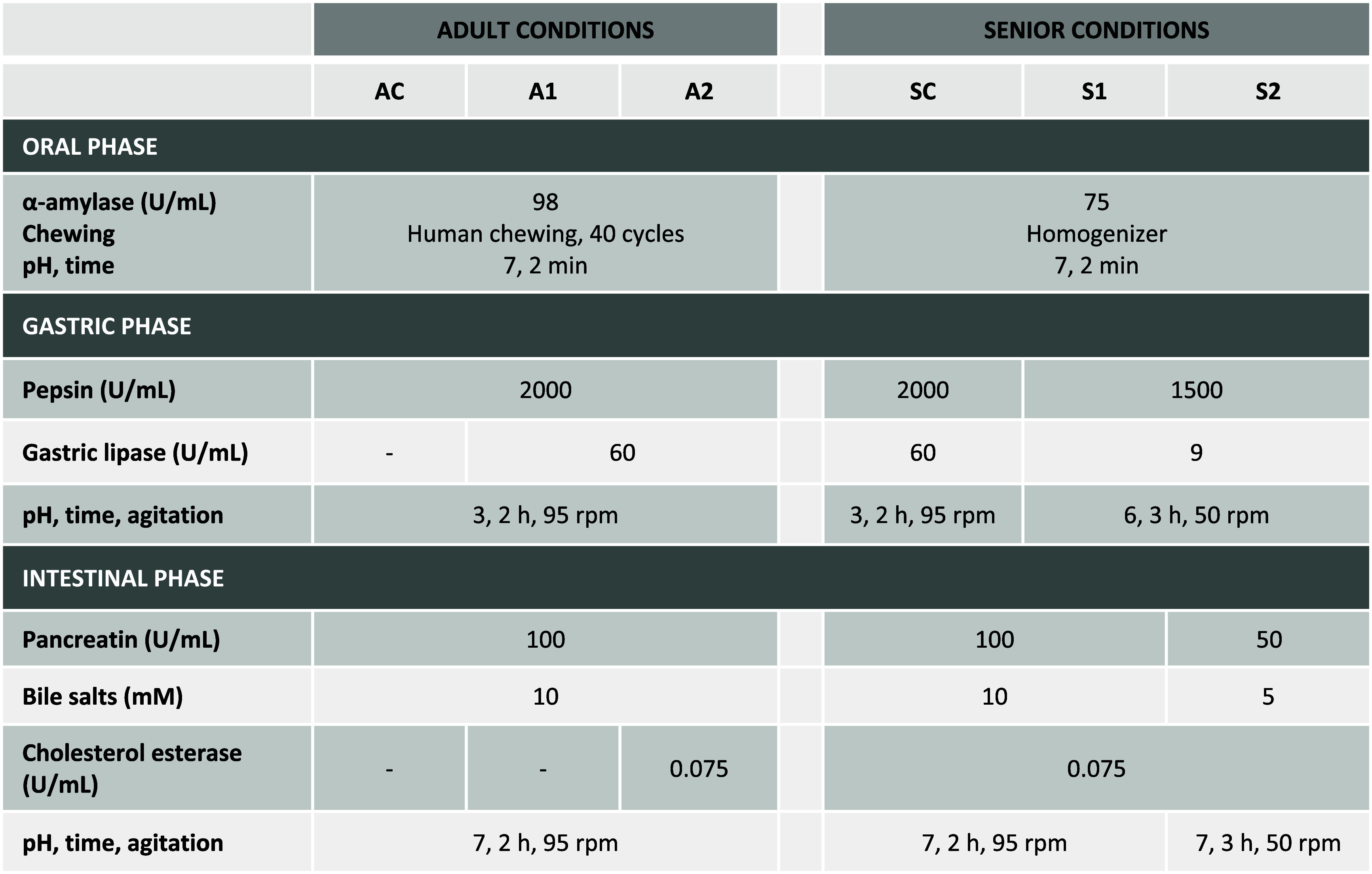
*In Vitro* Gastrointestinal Digestion Conditions for Adult and Senior Population Assays (AC: Adult Control; A1: Adult 1; A2: Adult 2; SC: Senior Control; S1: Senior 1; S2: Senior 2)

#### 2.3.1 Adult Condition

For adult condition, three different digestion methods were carried out: (i) adult control (AC) which corresponds to INFOGEST method;^16^ (ii) adult 1 (A1) which corresponds to INFOGEST 2.0 method;^18^ and (iii) adult 2 (A2) which corresponds to a modified version of the INFOGEST 2.0 method proposed by Makran et al.^17^

Briefly, a 5 g portion of fresh PS-WRB was subjected to 40 chewing cycles to simulate the oral phase. The gastric phase was mimicked by adding simulated gastric fluid and pepsin with GL (from rabbit gastric extract) included for A1 and A2 conditions. The intestinal phase was conducted by the addition of simulated intestinal fluid, pancreatin, and bovine bile extract. CE was also incorporated in A2 conditions, based on the enzyme activity provided by the manufacturer. The supernatant of the bioaccessible fraction was obtained by centrifugation at 3100*g* for 90 min at 4 °C (Eppendorf centrifuge 5810R, Hamburg, Germany). Digestions were carried out in triplicate, and the corresponding blanks of digestion were prepared in parallel to assess the contribution of the digestion reagents to the content of lipolysis compounds (FFA, MAG, DAG), PS and POPs.

#### 2.3.2 Senior Condition

Adaptation of digestion conditions to senior population was performed according to the modifications used in a previous study.^13^

To ensure a homogeneous digestion process, the mastication process for the senior condition was simulated by using a homogenizer. As a control digestion (senior control, SC), healthy adult conditions with the addition of GL and CE were used. An *in vitro* oral phase was employed, where partially dehydrated PS-WRB (3.7 g) was rehydrated with ultrapure water (1.3 g), mixed with simulated salivary fluid, and shaken. Adaptations for gastric (S1) and gastric-intestinal (S2) conditions involved changes in enzyme activities, pH, and agitation (Table 1). The corresponding bioaccessible fractions were obtained in the same way as previously indicated for digestion under adult conditions. Digestions were conducted in triplicate, and the corresponding blanks of digestion were also run.

### 2.4 Lipid Extraction

Lipid fractions from all samples were extracted using the Folch method^19^ with slight modifications. For total lipid profile determination, 10 g of flour or PS-WRB, 20 g of WRB, and 5 mL of bioaccessible fraction were first added with 5α-cholestane as IS (6 mg for flour, WRB, and PS-WRB samples, and 1 mg for bioaccessible fraction samples) and their lipids were extracted with chloroform:methanol (2:1, v/v) at 60 °C. After filtration, the samples were kept overnight at 4 °C with a 1 M KCl solution. The organic phase was then recovered, and the solvent was evaporated. The lipid phase was redissolved in *n*-hexane:isopropanol (4:1, v/v). Three independent replicates were carried out for each sample. The extraction of lipid fractions for the determination of PS and POPs from 20 g of flour and PS-WRB or 10 g of WRB was carried out following the same procedure but without adding 5α-cholestane, because the corresponding IS were directly added to the extracted lipids (see Section 2.6).

### 2.5 Total Lipid Profile of Flour, Bread, and Bioaccessible Fractions

Gas chromatography-flame ionization detection (GC-FID) was used to determine the qualitative-quantitative profile of the main lipid classes (FFA; MAG; tocopherols, TOC; free sterols, STE; diacylglycerols, DAG; esterified sterols, E-STE; triacylglycerols, TAG), as reported by Toschi et al.^20^ and Luise et al.^21^

An aliquot of 20 mg of the lipid extract diluted in 1 mL of *n*-hexane was injected in the GC-FID, using the conditions suggested by Toschi et al.^20^ The internal standard approach was used to calculate the amount of each lipid class (expressed as g/100 g of lipids) using the response factor of each lipid class (calculated with commercial standards).^21^ Three independent replicates were run for each sample.

### 2.6 Determination of Plant Sterols and POPs

#### 2.6.1 PS Ingredient, Flour, and Bread

For PS determination in flour and breads, lipid extracts (about 200 mg) were cold saponified with 400 μg of epicoprostanol as IS.^22^ The unsaponifiable fraction was subsequently extracted with diethyl ether and silylated as described by Inchingolo et al.^22^ and injected into a GC-MS under the same conditions as Cuevas-Tena et al.^23^

For POP determination, lipid extracts (about 300 mg) were added with 10 μg of 19-hydroxycholesterol as IS. The cold saponification and the extraction of the unsaponifiable fraction were carried out as mentioned above, but an additional, final step of POP purification and enrichment was run by silica solid-phase extraction (SPE).^24^ After silylation, POP content was determined using GC-MS under the same conditions indicated by Alemany et al.^24^

For the determination of PS and POPs in the PS ingredient, no lipid extraction was carried out and a direct cold saponification of 5 mg of ingredient was performed using the aforementioned methodology.^22,24^

The identification of PS and POP was performed based on mass fragment patterns of commercial standards and those reported in a previous study.^24^ Quantification was carried out using calibration curves obtained with commercial standards; for POPs, commercial standards of cholesterol oxidation products were employed. Three independent replicates were run for each sample.

#### 2.6.2 Bioaccessible Fractions

The bioaccessible fractions from all digestion conditions assayed were subjected to similar procedures to those described for PS and POP determination in Section 2.6.1, with slight differences.^24^ Briefly, 5 mL of bioaccessible fraction was taken, and 400 μg of epicoprostanol and 10 μg of 19-hydroxycholesterol (as IS for PS and POPs, respectively) were added. The sample was subjected to a direct cold saponification; the extracted unsaponifiable fraction was dissolved into 1 mL of *n*-hexane:isopropanol (4:1, v/v) and divided as follows: 300 μL for PS determination and 700 μL for POPs analysis. The latter was purified by SPE as reported in Section 2.6.1. Both PS and POPs were then silylated and injected into the GC-MS.^24^

The bioaccessibility of total and individual PS or POP was calculated according to the following formula: (PS or POP content in bioaccessible fraction ×100)/PS or POP content in bread.

### 2.7 Statistical Analysis

All experiments were performed in triplicate. The data are reported as mean values and standard deviations (SD). The Shapiro–Wilk method was used to test the normal distribution of data (*p* < 0.05). To distinguish statistically different means across the samples, a one-way analysis of variance (ANOVA) was performed, followed by Tukey’s honest significance test at a 95% confidence level (*p* < 0.05). A principal component analysis (PCA) with a varimax rotation was also used to assess data correlation. Software XL-STAT (7.5.2 version, Addinsoft, France) and GraphPad Prism v6.01 (GraphPad Software, La Jolla, CA) were used to analyze the data.

## 3 Results and Discussion

### 3.1 Ingredient, Flour, and Bread

#### 3.1.1 Total Lipid Profile

In both flour and WRB samples (Table 2), TAG was the most abundant lipid class, comprising 53.0 and 52.3% of total lipids, respectively. FFAs were the second most relevant component, accounting for 19.9% in flour and 13.8% in WRB. Other lipid classes present included STE, MAG, DAG, E-STE, and TOC, in varying proportions. The lipid profile of WRB closely resembled that of the flour used for its production. Higher values of DAG were found in WRB with respect to flour (8.9 *vs* 6.1% of total lipids), which could be attributed to a lipolytic phenomenon during the bread leavening due to the action of endogenous enzymes and/or those present in the yeast.^25^ Regarding the PS ingredient (Table 2), STE was the most abundant class (68.9% of total lipids), followed by DAG and MAG (15.9 and 15.3% of total lipids, respectively); the partial glycerides are used in this type of powder formulation to protect STE. Finally, regarding PS-WRB, as expected, STE was the most abundant lipid class (82.1% of total lipids), followed by TAG (12.3% of total lipids) (Table 2).

**Table 2:** Content of Lipids (%) and Main Lipid Classes (mg/100 g) Present in Flour and Bread Samples^a^^,^^b^

	flour	WRB	PS ingredient	PS-WRB
lipid content	2.81 ± 0.03^c^	1.20 ± 0.12^d^	3.40 ± 0.04^b^	4.53 ± 0.49^a^
FFA	182.28 ± 5.77 (19.92 ± 1.44)^a^	32.43 ± 1.95(13.77 ± 0.50)^b^	n.d.^d^	28.01 ± 0.74 (1.60 ± 0.04)^c^
MAG	64.01 ± 0.77 (6.99 ± 0.37)^b^	10.44 ± 0.57 (4.43 ± 0.17)^c^	14486.59 ± 5.60 (15.27 ± 0.76)^a^	10.39 ± 0.26 (0.59 ± 0.01)^d^
TOC	21.75 ± 0.86 (2.38 ± 0.19)^b^	7.21 ± 0.46 (3.06 ± 0.13)^a^	n.d.^c^	16.75 ± 0.67 (0.96 ± 0.04)^d^
STE	78.29 ± 0.04 (8.55 ± 0.35)^d^	28.60 ± 1.00 (12.15 ± 0.23)^c^	65327.22 ± 23.17 (68.86 ± 1.32)^b^	1436.38 ± 11.72 (82.08 ± 0.22)^a^
DAG	55.77 ± 4.93 (6.08 ± 0.29)^c^	20.87 ± 0.50 (8.87 ± 0.11)^b^	15055.80 ± 7.72 (15.87 ± 0.21)^a^	25.47 ± 1.16 (1.46 ± 0.06)^d^
E-STE	28.26 ± 2.37 (3.08 ± 0.13)^b^	12.83 ± 0.30 (5.45 ± 0.06)^a^	n.d.^d^	17.44 ± 0.15 (1.00 ± 0.00)^c^
TAG	486.06 ± 37.40 (53.00 ± 1.93)^a^	123.01 ± 1.77 (52.27 ± 0.91)^a^	n.d.^c^	215.62 ± 1.93 (12.32 ± 0.17)^b^
total	916.42 ± 37.26	235.40 ± 5.59	94869.61 ± 19.78	1750.06 ± 10.21

a Relative abundance is reported within parentheses.

b Data are expressed as mean ± standard deviation. Different letters (a–d) indicate significant differences (*p* < 0.05) between abundance percentage for each main lipid class. DAG, diacylglycerols; E-STE, esterified sterols; FFA, free fatty acids; MAG, monoacylglycerols; n.d., not detected; STE, sterols; TAG, triacylglycerols; TOC, tocopherols.

#### 3.1.2 Plant Sterols and Phytosterol Oxidation Products

As reported in Table 3, the PS ingredient had the highest phytosterol content (67/100 g of sample), characterized mainly by β-sitosterol (83.7%), followed by sitostanol (8.1%), and campesterol (5.3%); the content of the other sterols was <1%. A much lower PS content was observed in flour and WRB (0.06/100 and 0.02/100 g, respectively), with β-sitosterol as the most abundant compound (44.9–49.5%), followed by campesterol (12.0–12.3%), sitostanol (9.4–10.1%), and other 6 minor PS (each PS < 10%).

**Table 3:** Content of Plant Sterols (mg/100 g) and Their Oxidation Products (μg/100 g), and the Phytosterol Oxidation Ratio (%) of Raw Materials and Bread Samples^a^^,^^b^

	PS ingredient	flour	WRB	PS-WRB
plant sterols				
campesterol	3543.02 ± 162.88 (5.28 ± 0.24)^c^	6.93 ± 0.27 (11.99 ± 0.47)^a^	2.34 ± 0.05 (12.32 ± 0.24)^a^	107.61 ± 6.74 (6.76 ± 0.42)^b^
campestanol	629.53 ± 43.91 (0.94 ± 0.07)^d^	4.94 ± 0.08 (8.55 ± 0.15)^b^	1.94 ± 0.03 (10.23 ± 0.14)^a^	27.32 ± 0.97 (1.72 ± 0.06)^c^
stigmasterol	208.37 ± 3.02 (0.31 ± 0.004)^c^	3.60 ± 0.31 (6.23 ± 0.54)^b^	1.68 ± 0.03 (8.87 ± 0.17)^a^	12.74 ± 0.11 (0.80 ± 0.01)^c^
β-sitosterol	56151.59 ± 1536.15 (83.67 ± 2.29)^a^	28.61 ± 0.07 (49.50 ± 0.11)^b^	8.54 ± 0.60 (44.94 ± 3.13)^b^	1294.01 ± 62.14 (81.32 ± 3.91)^a^
sitostanol	5640.10 ± 400.63 (8.06 ± 0.09)^ab^	5.83 ± 0.62 (10.09 ± 1.07)^a^	1.78 ± 0.04 (9.35 ± 0.19)^a^	108.18 ± 6.63 (6.80 ± 0.42)^b^
Δ^5^-avenasterol	23.81 ± 1.99 (0.04 ± 0.003)^c^	1.94 ± 0.19 (3.35 ± 0.32)^b^	0.81 ± 0.02 (4.25 ± 0.09)^a^	0.64 ± 0.05 (0.04 ± 0.003)^c^
Δ^5,24^-stigmastadienol	315.47 ± 30.02 (0.47 ± 0.04)^b^	2.03 ± 0.17 (3.50 ± 0.30)^a^	0.75 ± 0.06 (3.94 ± 0.32)^a^	13.04 ± 1.10 (0.82 ± 0.07)^b^
Δ^7^-stigmastenol	543.15 ± 57.90 (0.81 ± 0.09)^b^	2.23 ± 0.20 (3.86 ± 0.35)^a^	0.73 ± 0.02 (3.82 ± 0.13)^a^	17.34 ± 1.25 (1.09 ± 0.08)^b^
Δ^7^-avenasterol	286.91 ± 30.31 (0.43 ± 0.05)^c^	1.69 ± 0.11 (2.92 ± 0.18)^a^	0.43 ± 0.03 (2.27 ± 0.16)^b^	10.35 ± 0.23 (0.65 ± 0.01)^c^
total	67112.01 ± 1507.75	56.97 ± 1.38	19.00 ± 0.68	1591.24 ± 76.57
phytosterol oxidation products				
7α-hydroxysitosterol	n.d.^b^	n.d.^b^	n.d.^b^	489.02 ± 55.88 (27.43 ± 1.08)^a^
7β-hydroxysitosterol	n.d.^c^	n.d.^c^	123.41 ± 2.32 (77.40 ± 1.05)^a^	450.11 ± 17.54 (25.32 ± 0.92)^b^
α-epoxysitosterol	n.d.^b^	n.d.^b^	n.d.^b^	510.44 ± 48.69 (28.65 ± 0.58)^a^
triol	n.d.^b^	n.d.^b^	n.d.^b^	123.32 ± 5.66 (6.96 ± 0.84)^a^
7-ketositosterol	n.d.^c^	n.d.^c^	36.07 ± 2.83 (22.60 ± 1.05)^a^	207.39 ± 17.31 (11.65 ± 0.10)^b^
total	n.d. ^c^	n.d.^c^	159.48 ± 5.15^a^	1780.28 ± 133.76^b^
β-sitosterol oxidation ratio			1.87^a^	0.14^b^

a Relative abundance is reported within parentheses.

b Data are expressed as mean ± standard deviation. Different letters (a–d) indicate statistically significant differences (*p* < 0.05) in each individual or total sterol abundance between samples (PS ingredient, flour, WRB, and PS-WRB).

The total PS content in the PS-WRB, instead, was 1.6/100 g, with the following relative distribution: β-sitosterol (81.3%) > campesterol and sitostanol (6.8%) > remaining PS (<2%). These results are comparable to those reported by Faubel et al.^12^ in PS-enriched rye bread samples.

Regarding POPs, they were not detected in the PS ingredient and in the flour, whereas only β-sitosterol oxides were found in both WRB and PS-WRB. WRB contained 159.48 μg of POPs/100 g of bread, with the most abundant POP being 7β-hydroxy derivative (77.4% of total POPs), followed by 7-keto (22.6% of total POPs). A higher content POPs content was found in PS-WRB (1780.28 μg POPs/100 g of bread), with the α-epoxy derivative being the most relevant (28.7% of total POPs), followed by 7α-hydroxy (27.4% of total POPs), 7β-hydroxy (25.3% of total POPs), 7-keto (11.7% of total POPs), and triol derivatives (7.0% of total POPs). While sterol oxidation products in WRB are mainly derived from the monomolecular reaction pathway (7-hydroxy and 7-keto derivatives), β-sitosterol oxides in PS-WRB are derived from both the bimolecular reaction pathway (5,6-epoxides, triol) and the monomolecular one.

There are few data in the literature about the amount of POPs in bakery products. Hu et al.^26^ determined the major dietary POPs in Chinese baked products and found that the content of total POPs ranged from 0.37 to 27.81 mg/g of products. These authors reported that the contribution of the chemical pathways to the formation of the main detected POPs was similar to those found in the present study. In general, Hu et al.^26^ reported that the POP concentration of cookies was higher than that of bread, which was attributed to distinct processing technologies and/or conditions, as well as their diverse specific surface area. Regarding the PS-oxidation ratio (OR), it was 1.9 and 0.1% in the WRB and PS-WRB samples, respectively. These values are far below those reported (2.2–12.8%) in the study by Hu et al.,^26^ probably because no oil was added into our bread and the chosen baking conditions (time and temperature) were less intense than those used by Hu et al.,^26^ thus leading to less oxidized samples.

### 3.2 Effect of Adult Digestion Conditions

#### 3.2.1 Lipid Profile

Table 4 shows the lipid profile of the bioaccessible fractions obtained under adult conditions. In all conditions assayed (AC, A1, and A2), FFA and STE were the main lipid class (43–47 and 46–51% of the total lipids, respectively), followed by MAG (3–4%), DAG, E-STE, and TAG (∼1%). A significant decrease in the abundance of STE (1.6- to 1.8-fold), DAG (1.4-fold), E-STE (2.5- to 3.5-fold), and TAG (10.0- to 14.5-fold) was observed, accompanied by a simultaneous increase in FFA (27.1- to 30.1-fold) and MAG (5.8- to 6.3-fold), when compared to the initial nondigested PS-WRB lipid profile. As expected, gastrointestinal *in vitro* digestion leads to the hydrolysis of bread TAGs, resulting in their conversion primarily into FFA and, to a lesser extent, into MAG.^27^

**Table 4:** Content of Lipids (%) and Main Lipid Classes (mg/100 g Bread) Present in Bioaccessible Fractions from *In Vitro* Digestion of PS-WRB under Adult and Senior Conditions^a^^,^^b^

	AC	A1	A2	SC	S1	S2
lipid content	0.17 ± 0.02^a^	0.15 ± 0.00^a^	0.16 ± 0.00^a^	0.16 ± 0.00^a^	0.16 ± 0.02^a^	0.16 ± 0.01^a^
FFA	227.59 ± 12.97^a^ (43.41 ± 1.07)^b^	213.62 ± 3.73^ab^ (48.12 ± 1.01)^a^	198.34 ± 5.00^b^ (46.55 ± 0.86)^a^	140.25 ± 5.12^a^ (43.17 ± 0.79)^a^	151.17 ± 2.99^a^ (48.18 ± 2.87)^a^	84.61 ± 3.27^b^ (45.63 ± 3.87)^a^
MAG	18.58 ± 1.60^a^ (3.39 ± 0.11)^a^	16.40 ± 1.01^ab^ (3.69 ± 0.13)^a^	14.85 ± 0.21^b^ (3.50 ± 0.02)^a^	11.07 ± 0.48^b^ (3.48 ± 0.07)^a^	13.24 ± 0.84^a^ (3.71 ± 0.21)^a^	8.99 ± 0.68^c^ (4.14 ± 0.37)^a^
STE	258.23 ± 15.93^a^ (51.03 ± 1.09)^a^	202.28 ± 10.16^b^ (45.53 ± 0.83)^b^	203.01 ± 2.65^b^ (47.66 ± 0.78)^b^	166.40 ± 11.86^a^ (51.18 ± 0.84)^a^	171.31 ± 13.67^a^ (48.06 ± 4.16)^a^	98.29 ± 4.72^b^ (47.16 ± 3.28)^a^
DAG	5.42 ± 0.15^a^ (1.02 ± 0.04)^a^	4.58 ± 0.09^b^ (1.03 ± 0.02)^a^	4.42 ± 0.08^b^ (1.04 ± 0.01)^a^	3.28 ± 0.04^b^ (1.01 ± 0.04)^b^	3.83 ± 0.16^a^ (1.07 ± 0.06)^b^	2.73 ± 0.12^c^ (1.33 ± 0.10)^a^
E-STE	1.61 ± 0.15^ab^ (0.29 ± 0.00)^b^	1.77 ± 0.14^a^ (0.40 ± 0.02)^a^	1.36 ± 0.07^b^ (0.32 ± 0.02)^b^	1.14 ± 0.08^a^ (0.34 ± 0.01)^ab^	1.12 ± 0.06^a^ (0.31 ± 0.02)^b^	0.77 ± 0.05^b^ (0.37 ± 0.01)^a^
TAG	4.53 ± 0.17^b^ (0.85 ± 0.06)^b^	5.48 ± 0.44^a^ (1.23 ± 0.08)^a^	4.04 ± 0.38^b^ (0.95 ± 0.10)^b^	2.63 ± 0.23^a^ (0.82 ± 0.05)^b^	2.61 ± 0.32^a^ (0.73 ± 0.04)^b^	2.94 ± 0.15^a^ (1.37 ± 0.11)^a^
total	524.05 ± 16.93^a^	444.14 ± 14.49^b^	426.01 ± 3.71^b^	325.01 ± 17.82^a^	343.60 ± 16.25^a^	185.54 ± 8.98^b^

a The relative abundance of main lipid classes is reported within parentheses.

b Data are expressed as mean ± standard deviation. Different letters (a–c) indicate significant differences (*p* < 0.05) in each lipid class between adult or senior modifications. AC, adult control; A1, adult 1; A2, adult 2; DAG, diacylglycerols; E-STE, esterified sterols; FFA, free fatty acids; MAG, monoacylglycerols; SC, senior control; S1, senior 1; S2, senior 2; STE, sterols; TAG, triacylglycerols.

Regarding the addition of different digestion enzymes, a significant increase in the TAG content (from 4.5 to 5.5/100 g) under A1 conditions (with the addition of GL) was observed. Despite this slight increase in the TAG content, no significant differences in the contents of lipolysis products (FFA and MAG) were observed between digestions with and without GL (A1 and AC, respectively). The limited GL contribution observed in our study can be due to diverse factors, such as the role of GL in the overall lipolytic process. GL-mediated TAG hydrolysis is minor compared to intestinal lipolysis facilitated by pancreatic lipase.^28^ However, the release of FFA at the stomach level plays a crucial role in promoting the secretion of bile and pancreatic juice as well as in potentiating subsequent pancreatic lipase activity. Indeed, the contribution of GL to TAG hydrolysis in solid meals has been estimated to be 10%,^14^ which could justify the lack of lysis effect observed when this enzyme was added during the digestion of our bread samples. Moreover, fiber content might also have contributed to limit the lipolytic effect of GL. In this regard, it has been demonstrated that the presence of soluble fiber at increasing concentrations in a lipid mixture resulted in larger lipid droplets and hindered the access of GL to TAG, possibly due to the increased viscosity caused by the soluble fiber.^29^

When both GL and CE were added in the bread digestion under A2 conditions, the only difference observed with respect to A1 (without CE) was an improvement in the hydrolysis of both E-STE and TAG. As expected, the nonspecificity of the CE enzyme led to the hydrolysis of both E-STE and TAG.^27,30^ This confirms that CE plays an additional role in the lipolytic enzymatic activity along with GL.

#### 3.2.2 Plant Sterols

Table 5 reports the PS content in bioaccessible fractions and their corresponding bioaccessibilities for all of the digestion methods conducted under adult conditions. Total PS content in bioaccessible fractions ranged from 224.8 to 402.0 mg/100 g of bread. For the most abundant PS (campesterol, campestanol, β-sitosterol, and sitostanol), a reduction of their contents in bioaccessible fraction was observed with the addition of lipid digestion-related enzymes (A2 > A1). The bioaccessibility of total PS was significantly reduced under A1 and A2 digestion conditions (18.6 and 14.1%, respectively), compared to the control digestion AC (25.3%). Regarding the PS solubility profile, sitostanol was the most bioaccessible PS in all of the digestion conditions assayed (21.3 and 32.8%), and Δ^7^-avenasterol was the lowest one (7.9–15.2%). These findings are in line with a previous work on similar samples of wholemeal rye bread digested under different INFOGEST conditions.^12^ The inclusion of GL or GL and CE resulted in a decrease in the bioaccessibility of PS in the bread (1.3- and 1.4-fold *vs* control digestion, respectively). Interestingly, bioaccessibility values reported by Faubel et al.^12^ were comparable to our results, despite using different methodologies for PS determination: INFOGEST method (23.8%) *vs* AC (25.3%); INFOGEST 2.0 method (18.5%) *vs* A1 (18.6%); INFOGEST 2.0 with CE (17.1%) *vs* A2 (14.1%). This fact confirms the consistency of the results across different experimental approaches. In the work by Faubel et al.,^12^ samples were subjected to an acid hydrolysis step with HCl (80 °C, 1 h) and a hot alkaline saponification to improve the release of PS from their glycoside and ester forms. This methodology was not used in the present study since one of our objectives was to determine the content of POPs. The use of acidic conditions and high temperatures could potentially lead to oxidation of the sterols during sample treatment, thus giving rise to artifact formation and overestimation of POPs in the bread samples. The similar total PS bioaccessibility values obtained in both works, together with the low abundance of E-STE determined in the present study (<1% in the PS-WRB and <0.4% in their corresponding bioaccessible fractions) suggest that acid hydrolysis and hot saponification steps previously employed by the authors would not significantly affect PS determination in our matrix.

**Table 5:** Contents of Sterols (mg/100 g Bread) and Sterol Oxidation Products (μg/100 g) in Bioaccessible Fractions, and Bioaccessibility (%) after *In Vitro* Digestions of PS-WRB for Adult and Senior Conditions^a^

	AC		A1		A2		SC		S1		S2	
	BF	BA	BF	BA	BF	BA	BF	BA	BF	BA	BF	BA
Plant Sterols (mg/100 g Bread)												
campesterol	20.83 (0.92)^a^	19.35 (0.86)^a,CDE^	17.37 (0.01)^b^	16.14 (0.01)^b,CD^	12.40 (0.21)^c^	11.52 (0.19)^c,D^	9.84 (0.79)^b^	9.14 (0.74)^b,C^	14.10 (0.58)^a^	13.10 (0.54)^a,B^	9.85 (0.11)^b^	9.15 (0.10)^b,C^
campestanol	5.41 (0.21)^a^	19.79 (0.75)^a,CD^	4.43 (0.11)^b^	16.20 (0.40)^b,CD^	3.18 (0.08)^c^	11.62 (0.30)^c,D^	2.68 (0.24)^a^	9.80 (0.89)^a,C^	3.44 (0.29)^a^	12.57 (1.07)^a,B^	2.73 (0.03)^a^	9.98 (0.12)^a,C^
stigmasterol	2.77 (0.06)^a^	21.76 (0.46)^a,BC^	2.50 (0.20)^ab^	19.58 (1.54)^ab,B^	2.10 (0.14)^b^	16.52 (1.06)^b,B^	1.51 (0.13)^b^	11.84 (1.06)^b,BC^	1.82 (0.04)^ab^	14.32 (0.28)^ab,B^	1.98 (0.05)^a^	15.55 (0.38)^a,A^
β-sitosterol	330.50 (3.93)^a^	25.54 (0.30)^a,B^	234.36 (6.23)^b^	18.11 (0.48)^b,BC^	179.97 (6.07)^c^	13.91 (0.47)^c,C^	147.42 (8.32)^b^	11.39 (0.64)^b,BC^	204.42 (13.69)^a^	15.80 (1.06)^a,B^	121.38 (3.62)^b^	9.38 (0.28)^b,C^
sitostanol	35.44 (1.46)^a^	32.76 (1.35)^a,A^	30.83 (0.37)^b^	28.50 (0.34)^b,A^	22.99 (0.61)^c^	21.26 (0.56)^c,A^	19.97 (1.74)^b^	18.46 (1.60)^b,A^	25.01 (0.10)^a^	23.12 (0.10)^a,A^	17.92 (0.61)^b^	16.56 (0.57)^b,A^
Δ^5^-avenasterol	0.13 (0.01)^a^	20.29 (1.86)^a,CD^	0.13 (0.001)^a^	19.47 (0.12)^a,B^	0.10 (0.0002)^a^	15.94 (0.04)^a,B^	0.09 (0.01)^a^	13.96 (1.59)^a,B^	0.09 (0.01)^a^	14.44 (1.23)^a,B^	0.08 (0.001)^a^	12.98 (0.12)^a,B^
Δ^5,24^-stigmastadienol	2.21 (0.19)^a^	16.98 (1.47) ^a,DE^	2.46 (0.08)^a^	18.85 (0.63)^a,B^	1.34 (0.05)^b^	10.27 (0.38)^b,D^	1.16 (0.01)^b^	8.88 (0.09)^b,C^	1.95 (0.17)^a^	14.96 (1.30)^a,B^	1.44 (0.11)^b^	11.06 (0.86)^b,BC^
Δ^7^-stigmastenol	3.14 (0.27)^a^	18.10 (1.58)^a,CDE^	2.52 (0.02)^ab^	14.50 (0.12)^ab,D^	1.86 (0.01)^b^	10.75 (0.04)^b,D^	1.57 (0.14)^b^	9.07 (0.80)^b,C^	2.55 (0.24)^a^	14.71 (1.38)^a,B^	1.79 (0.07)^b^	10.30 (0.41)^b,C^
Δ^7^-avenasterol	1.57 (0.06)^a^	15.15 (0.54)^a,E^	1.50 (0.08)^a^	14.45 (0.78)^a,D^	0.81 (0.06)^b^	7.87 (0.56)^b,E^	0.93 (0.06)^b^	8.99 (0.55)^b,C^	1.40 (0.12)^a^	13.55 (1.13)^a,B^	1.08 (0.11)^ab^	10.40 (1.02)^ab,C^
total	402.00 (1.36)^a^	25.26 (0.09)^a^	296.07 (6.28)^b^	18.61 (0.39)^b^	224.77 (6.64)^c^	14.13 (0.42)^c^	185.17 (5.20)^b^	11.64 (0.33)^b^	254.79 (12.36)^a^	16.01 (0.78)^a^	158.24 (4.12)^b^	9.94 (0.26)^b^
Phytosterol Oxidation Products (μg/100 g Bread)												
7α-hydroxysitosterol	87.26 (0.36)^b^	17.84 (0.07)^b^	109.42 (3.21)^a^	22.38 (0.66)^a^	106.97 (4.25)^a^	21.87 (0.87)^a^	125.51 (4.15)^b^	25.67 (0.85)^b^	148.72 (6.17)^a^	30.41 (1.26)^a^	98.40 (0.97)^c^	20.12 (0.20)^c^

a Data are expressed as mean and standard deviation (between parentheses). Different letters (a-c) indicate statistically significant differences (*p* < 0.05) in each sterol between bioaccessible fraction content or bioaccessibility of each modification for adult or senior assays. Different letters (A-E) indicate statistically significant differences (*p* < 0.05) between individual plant sterol bioaccessibility for each digestion condition (adult and senior assays). AC, adult control; A1, adult 1; A2, adult 2; BA, bioaccessibility; BF, bioaccessible fraction; SC, senior control; S1, senior 1; S2, senior 2.

The decrease in PS bioaccessibility by the addition of GL or GL and CE during adult *in vitro* digestion has also been reported in a PS-enriched beverage.^17^ These authors suggested that the addition of these enzymes enhances lipid digestion and, thus, FFA, MAG, and DAG production as a result of TAG hydrolysis. These lipolysis products play a crucial role in the disruption of lipid droplets,^31^ as well as in the number and size of mixed micelles,^32^ and therefore in their PS solubilization capacity; in fact, the hydrophobic bioactive molecules must be small enough to fit into the hydrophobic core of micelles.^36^ Therefore, Makran et al.^17^ suggested that their formation may facilitate the incorporation of cholesterol from both the analyzed beverage and the digestion reagents into the mixed micelles. Consequently, the incorporation of PS is reduced by a competition process with cholesterol, which also results in a reduction in its solubility. Our findings partially support this hypothesis, as an increase in the abundance of FFA occurred when specific lipid metabolism enzymes were added during digestion. However, analysis of the lipid profile of the samples did not show an increase in the abundance of STE, which comprises both PS and cholesterol compounds. The cholesterol content from the digestion blanks was subtracted from the contents measured in the bioaccessible fractions of the samples, following an approach similar to that of the PS determination. The results indicated that the cholesterol determined in the bioaccessible fractions (only provided by the digestion reagents) remains constant under all digestion conditions (AC, A1, and A2) (data not shown) since a negligible amount of cholesterol is provided by the bread. Therefore, the competition between cholesterol and PS previously reported by Makran et al.^17^ depends on the amount of cholesterol present in the analyzed matrix; in fact, such competition did not take place in our study as cholesterol was not present in our samples and it was only provided by the reagents. In contrast, the incorporation of specific enzymes involved in lipid metabolism, as described in Section 3.2.1, leads to a progressive decrease of all lipolysis products (FFA, MAG, and DAG). These data suggest that the reduction in PS solubility could be more likely attributed to the decrease in lipolysis products than to the preferential inclusion of cholesterol in mixed micelles.

Overall, Makran et al.^17^ confirmed that the incorporation of GL and CE to the digestion method proposed by Minekus et al.^16^ provides a more realistic approximation of *in vivo* gastrointestinal conditions. Specifically, bioaccessibility values for total PS, stigmasterol, and campesterol obtained *in vitro* by Makran et al.^17^ with the combined addition of GL and CE (8, 4.8, and 9.6%, respectively) closely resembled human absorption rates (6, 5.5, and 10.9%, respectively) previously reported.^33,34^ Similarly, our study demonstrated a closer approximation to these values when conducting PS-WRB digestion in the presence of GL and CE (A2 condition) than without their addition (AC condition). Bioaccessibility values for total PS, stigmasterol, and campesterol were notably lower with the inclusion of GL and CE (14.1, 16.5, and 11.5%, respectively) than without them (25.3, 21.8, and 19.4%, respectively). This suggests that the A2 condition better reflects the realistic bioavailability of these compounds during digestion, reinforcing the importance of incorporating specific enzymes of lipid metabolism for a more accurate *in vitro* simulation of physiological conditions.

#### 3.2.3 Phytosterol Oxidation Products

From the analysis of POPs contents in the bioaccessible fractions at adult digestion conditions, only 7α-hydroxysitosterol was detected (Table 5). Its content ranged from 87.3 μg/100 g in AC samples to 109.4 and 107.0 μg/100 g under A1 and A2 conditions, respectively.

To the best of our knowledge, only two studies have determined the bioaccessibility of POPs after an *in vitro* digestion of PS-enriched foods (milk and milk-based fruit beverages).^24,35^ Likewise in our study, only β-sitosterol oxides were detected, as it is the most abundant PS in the ingredient used for food enrichment. However, they identified other sterol oxides after gastrointestinal digestion such as 7β-hydroxy, α/β-epoxy, triol, and 7-keto. In fact, in these studies, the relative percentage of 7α-hydroxy was lower compared with the other derivatives. Differences in the profile of β-sitosterol oxides could be attributed to differences in the digestion methods. Alemany et al.^24^ and Alvarez-Sala et al.^35^ employed a digestion protocol which included the addition of specific enzymes of lipid metabolism (pancreatic lipase, colipase, CE, and phospholipase A2). However, this protocol has shortcomings, which have been highlighted as crucial within the harmonized INFOGEST method^16^ and its subsequent updating (INFOGEST 2.0).^18^ Among them, the standardization of the synthetic fluids added in the oral, gastric, and intestinal phases, as well as the pH and time conditions of the three digestive stages, is based on physiological parameters. Moreover, the addition of digestive enzymes (based on their enzymatic activity) and bile extracts (based on their bile acid content) adopted in the INFOGEST method represents an approach to physiological conditions, which is not addressed in other digestion methods, thus probably being the key point for the different results obtained.

In addition, the higher bioaccessibility of 7α-hydroxysitosterol reported in the present work after the INFOGEST digestion method is consistent with *in vivo* studies in which 7α- and β-hydroxy derivatives showed higher absorption ratios compared to other sterol oxides.^36,37^

Regarding total POP contents in bioaccessible fractions, the values obtained in the present study are higher than those reported by Alemany et al.^24^ (19–33 μg/100 g beverage), but similar to those observed by Alvarez-Sala et al.^35^ (86–93 μg/100 g beverage), despite the higher total POPs content in the undigested bread samples (187–204 μg POPs/100 g beverage *vs* 1780 μg POPs/100 g bread). In this regard, the higher fiber content of bread (with respect to that of beverages) might have potentially led to a lower solubilization of POPs. In fact, a recent study reported that the addition of oat fiber (0.8%) to a high-fat and high-cholesterol diet was able to decrease the content of cholesterol oxides in the plasma of mice.^38^

As shown in Table 5, POPs bioaccessibility significantly increased with the addition of GL (A1, 22.4%), and GL and CE (A2, 21.9%) *vs* control conditions (AC, 17.8%). Despite the reduction of lipolysis products with the addition of specific lipid metabolism enzymes, the higher abundance of these emulsifying compounds in A1 and A2 might have favored the incorporation of POPs, rather than PS, into the bile salt micelles. In addition, the chemical structure of POPs, including functional groups such as hydroxyl, makes them more polar compared to nonoxidized sterols and therefore more soluble in aqueous environments like the intestinal medium.^39^

POPs showed higher bioaccessibilities than PS under A1 and A2 conditions, displaying an opposite trend under AC conditions. The higher bioaccessibility of POPs compared to PS was also observed by Alemany et al.^24^ (2–7% for PS *vs* 19–49% for POPs). Although *in vivo* studies have demonstrated an increase in serum POPs levels in response to PS-enriched diets,^40^ studies providing information on the absorption ratios of POPs *vs* PS are scarce. However, in line with the results obtained by *in vitro* digestions (Alemany et al.^24^ and the present work), a study conducted in thoracic duct-cannulated rats^36^ reported higher lymphatic recoveries of campesterol and β-sitosterol oxides compared to their nonoxidized sterols (16 *vs* 6% and 9 *vs* 2%, respectively). These results suggest that the inclusion of specific lipid metabolism enzymes during *in vitro* digestion improves the *in vitro*–*in vivo* correlation.

### 3.3 Effect of Senior Digestion Adaptations

The inclusion of enzymes involved in lipid metabolism, such as GL and CE, led to a reduction in PS bioaccessibility and an increase in POPs bioaccessibility, as discussed in Sections 3.2.2 and 3.2.3. Since the incorporation of these enzymes provides a more accurate assessment of PS and POPs bioaccessibility under physiological conditions, A2 digestion (which includes GL and CE) was thus chosen as the control digestion (SC) to evaluate the impact of digestion modifications in senior population.

#### 3.3.1 Lipid Profile

Table 4 reports the lipid profile in the bioaccessible fraction samples obtained from the adaptations of the digestions to senior population conditions. In terms of abundance, no significant differences were observed in the lipid profiles between SC and S1 digestions. However, a significant increase in the content of MAG and DAG (1.2-fold) was observed in the adaptation of the gastric phase to senior conditions; a slight but no significant increment of the FFA content was also noted (from 140 mg/100 g for SC to 150 mg/100 g with S1 modifications). The main modification in this phase was the drastic reduction of GL activity (from 60 to 9 U/mL) along with the increase in pH to 6 (far from the optimal pH for this enzyme). These results are in line with those observed for the digestion conditions in adults (Section 3.2.1), where the inclusion of GL resulted in only slight changes in the content of lipolysis products. Furthermore, the extended duration of the gastric phase under S1 conditions (compared to SC) may explain the observed tendency toward increased levels of partial (DAG, MAG) and complete lipolysis products (FFA). In a recent study, Hernández-Olivas et al.^41^ reported that gastric conditions simulating those of the elderly population did not notably affect chia seed digestibility. Likewise our results, the authors justified the lack of impact on digestibility by suggesting that the unmodified intestinal phase could have compensated the changes produced by the modified gastric phase. Digestion under S2 conditions resulted in a significant increase in the abundance of DAG (32%), E-STE (9%), and TAG (67%) with respect to SC, even though the content of all main lipid classes decreased significantly compared with SC and S1. No significant differences in the TAG content among the digestion conditions were found. These results demonstrate a decrease in lipid digestion efficiency under senior population conditions. In this context, studies have shown contradictory effects of elderly digestion conditions on lipolysis when different food products were evaluated.^17^ While dairy products and poached eggs exhibited increased lipolysis under senior adult conditions compared to adult digestion conditions, hard-boiled eggs showed a decreased hydrolysis extent and no significant changes were observed for salmon or sea bass. However, senior adult digestion conditions for chia seeds resulted in a significant decrease of lipid digestion,^41^ which is in line with our findings. In this sense, the higher content of fiber in chia seed (30/100 g) and in the bread here evaluated (20/100 g) compared to the above-mentioned animal-based products could justify this lower lipid digestion activity in the adapted gastric phase for senior population conditions. Zhou et al.^42^ demonstrated that the extent of lipid digestion in plant-based beef was significantly lower compared to the beef control sample. The presence of dietary fibers seems to inhibit lipid digestion by trapping some oil droplets or interacting with gastrointestinal substances such as bile salts and lipase. Therefore, it is likely that in fiber-rich products, the specific digestive conditions of the senior population, characterized by lower pancreatic lipase activity and reduced levels of bile salts, have a detrimental impact on lipid digestion that cannot be compensated by a prolonged intestinal transit time.

#### 3.3.2 Plant Sterols

Table 5 shows the impact of S1 and S2 adaptations on the content of individual and total PS in the bioaccessible fraction, as well as their corresponding bioaccessibilities compared to SC.

Under S1 conditions, a significant increase in the solubility of total PS (1.4-fold) compared to that of SC was observed. The solubility profile is partially maintained with sitostanol being the most bioaccessible PS (23.1%), as in the SC; however, no significant differences were observed in the bioaccessibilities of the other PS (12.6–16.0%). The increase of PS bioaccessibility (from 11.6% in SC to 16.0%) in S1 adaptation (with reduced GL activity and increased gastric pH) is consistent with the higher bioaccessibility observed in AC (without GL) *vs* A1 (with GL) conditions. In fact, similar reductions of approximately 1.4-fold in total PS bioaccessibility were observed when comparing AC *vs* A1 digestions, as well as when confronting digestions with reduced GL activity and suboptimal pH conditions, to the control method under senior adult conditions (S1 *vs* SC). The consistent results across different digestion conditions highlight the significant impact of GL on the solubilization of PS, ultimately influencing their overall bioaccessibility, despite no significant effects were observed in the analysis of the main lipid classes.

When both gastric and intestinal conditions were adapted to the senior population (S2), a significant shift in the solubility profile of PS was evident compared to SC, with sitostanol and stigmasterol emerging as the most bioaccessible sterols (16.6 and 15.6%, respectively), in contrast to only sitostanol. However, both the content and bioaccessibility of total PS did not show significant differences compared to SC. It seems that the increase in PS bioaccessibility caused by the reduction in GL activity by S1 modification is offset by the reduction in pancreatin activity and bile salt concentration in the S2 modification. In fact, the negative impact on lipid digestion caused by reduced pancreatic lipase activity and reduced bile salt concentration resulted in a minor PS solubilization compared to the modification of the gastric phase alone.

In a previous study, PS bioaccessibility in a PS-enriched milk-based fruit beverage^43^ was evaluated under senior *in vitro* digestion conditions. In the beverage, in contrast with our results, the specific gastrointestinal conditions of the elderly significantly increased the bioaccessibility of PS compared to adult conditions (15 *vs* 8%). The authors indicated that the reduction of bile and pancreatin diminished the cholesterol content provided by these digestion reagents, which facilitated the solubilization of the sterols provided by the beverage (cholesterol and PS). In another study,^13^ the implementation of senior digestion conditions for the evaluation of PS bioaccessibility on PS-enriched bread samples demonstrated partial agreement with our findings. On the one hand, Miedes et al.^13^ reported that implementing the gastric phase under senior conditions did not have an impact on PS bioaccessibility in bread samples. The authors attributed the lack of effect to the minimal contribution of GL to TAG hydrolysis in solid foods. However, our study revealed that despite the overall similarity in lipid profile between SC and S1 digestion conditions, modifications in the gastric phase can result in a significant increase in MAG content and a slight increase in FFA. The slight increase of these lipolysis products might be responsible for the enhanced solubility of PS compared to the control method since, as mentioned above, they are involved in the solubilization capacity of the mixed micelles. On the other hand, and like our findings, Miedes et al.^13^ observed that adapting both the gastric and intestinal phases to senior conditions decreases the bioaccessibility of PS compared to adapting only the gastric phase. In this regard, as mentioned above, the reduction of pancreatic lipase and bile content has a significant impact on lipid digestion, particularly on sterols, which can be further exacerbated by the presence of dietary fiber.

#### 3.3.3 Phytosterol Oxidation Products

In digestions carried out under senior conditions, as previously observed for adult conditions, the only POP identified was 7α-hydroxysitosterol. As shown in Table 5, under S1 modification, there was an increase in the content of 7α-hydroxysitosterol in bioaccessible fractions from 125.5 to 148.7 μg/100 g of bread. However, when the gastric modification was combined with the intestinal modification (S2 condition), a significant reduction was observed, with values reaching 98.4 μg/100 g of bread. Similarly, the bioaccessibility of 7α-hydroxysitosterol in these digestions was 25. 7% for SC, 30.4% for S1, and 20.1% for S2. These results are in line with those found for PS (Section 3.3.2), since the modification S1 increased the bioaccessibility of PS and POPs with respect to SC, whereas S2 decreased them. In S1, a similar content of FFA is observed compared to the control method which, along with a longer duration of this stage, could have favored the POPs solubilization. On the other hand, the modifications in S2 digestion, which resulted in a significant decrease of the lipolysis products (Section 3.3.1), led to a reduction of POPs solubility, as for PS.

To our knowledge, no previous studies have evaluated the effect of adapting *in vitro* gastrointestinal digestion to senior conditions on the bioaccessibility of POPs. The effect of POPs on the initiation and progression of various pathologies has gained interest in recent decades but is still scarce. In this regard, it has been observed that 7β-hydroxy and 7-keto derivatives of β-sitosterol show a greater cytotoxic potential, and that β-sitosterol oxides generate a greater induction of cell apoptosis than those deriving from campesterol and stigmasterol.^44,45^ The role of POPs in inflammatory processes remains inconclusive, as some studies have reported no effect on proinflammatory cytokine secretion,^46,47^ while others have observed an increase.^48^ Regular dietary intake of POPs does not lead to an increase in atherosclerotic lesion size in mice,^47^ but the decrease in aortic functionality observed in hamsters and rats suggests that they may have a potential atherogenic effect.^37^ In this sense, the cytotoxicity induced by POPs could become more accentuated with age, as with cholesterol oxides. Several age-related diseases, such as Alzheimer’s disease or cardiovascular diseases, have been associated with increased levels of 7-ketocholesterol and 7β-hydroxycholesterol in plasma and/or tissues.^49,50^ Therefore, further research is required to better understand the underlying mechanisms and potential implications of our findings for the nutritional assessment and health implications of POPs in the senior population.

## 4 Principal Component Analysis

**Figure 1:**
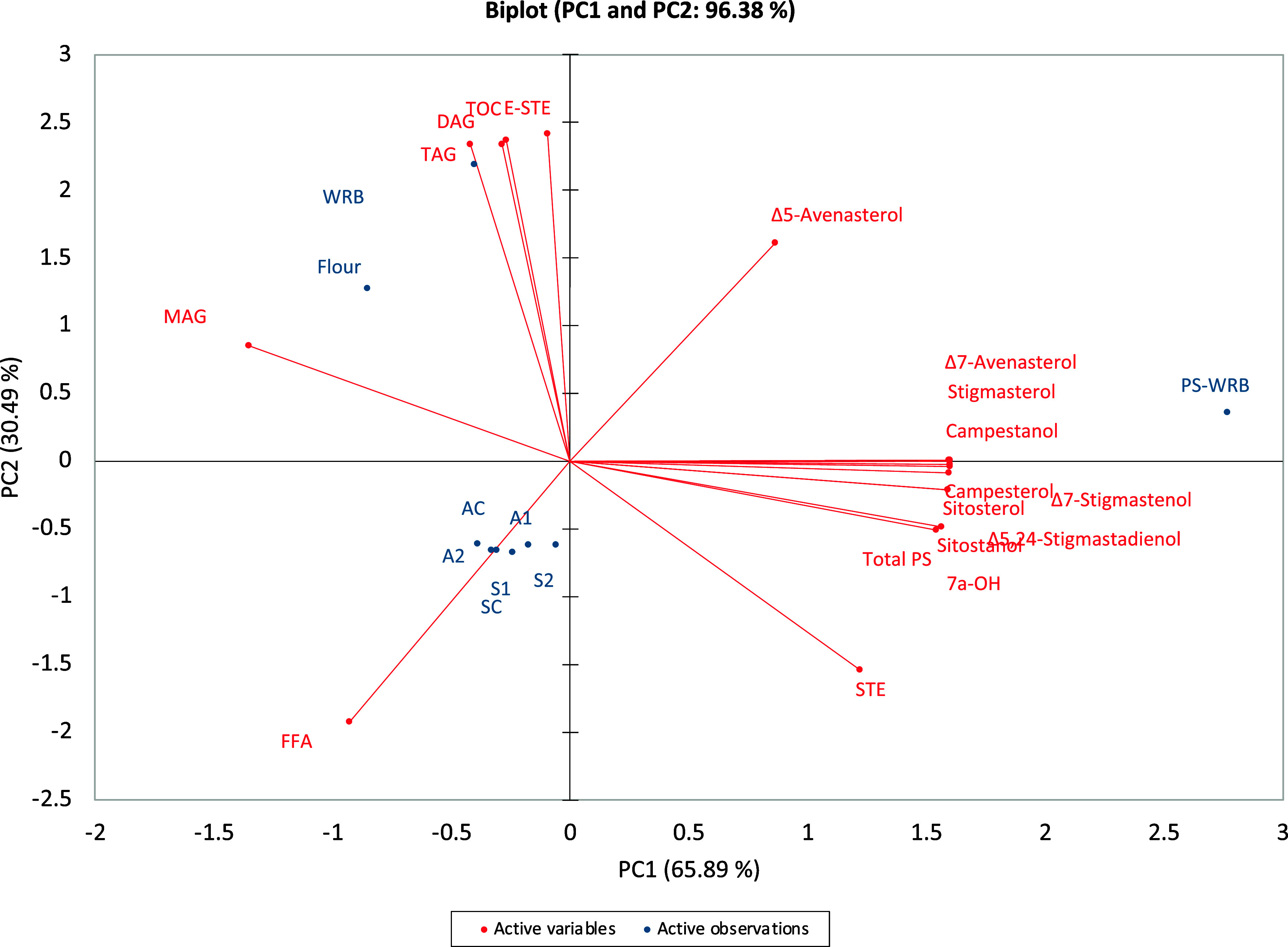
Biplot of all parameters. AC, adult control; A1, adult 1; A2, adult 2; DAG, diacylglycerols; E-STE, esterified sterols; FFA, free fatty acids; MAG, monoacylglycerols; PS, plant sterols; PS-WRB, PS-enriched wholemeal rye bread; SC, senior control; S1, senior 1; S2, senior 2; STE, sterols; TAG, triacylglycerols; TOC, tocopherols; WRB, wholemeal rye bread.

To better understand the correlations between the different parameters and how changes in *in vitro* digestion impacted the total lipid profile and the distribution of total sterols and their oxidation products, all data were subjected to PCA (Figure 1).

The first two components explained 96.4% of the total variance (65.9% for PC1 and 30.5% for PC2). As depicted in Figure 1, there are 3 distinct clusters, of which the first, located in quadrant 2, includes the flour and WRB samples that are correlated with the main lipid classes (TAG, DAG, MAG, TOC, and E-STE), except for STE. The second cluster, located in quadrant 1, comprises the PS-WRB sample, which is correlated with the STE variable and all identified sterols and POPs; this was somehow expected considering the composition of the sterol-enriching ingredient. Finally, the third cluster, located in quadrant 3, includes the *in vitro* digestion samples, which are characterized by the FFA variable; this demonstrates that the formation of FFA during lipid digestion is crucial in influencing the amount and size of mixed micelles produced, and thus their PS solubilization potential.^31,32^ As a result, their release from glyceridic molecular structures may enhance the incorporation of cholesterol into the mixed micelles, but further research is needed to fully clarify this mechanism.

In summary, the present study reveals that adding PS to wholemeal rye bread significantly increased POPs content after baking, evidencing distinct POP formation pathways between WRB and PS-WRB. These findings are crucial for WRB characterization and understanding digestion’s impact on its lipid fraction. To assess the impact of *in vitro* digestion on lipolysis, PS oxidative stability, and bioaccessibility of PS-WRB, different INFOGEST digestion conditions were used to mimic those of adults and elderly population. Modifications with specific lipid metabolism enzymes during adult conditions reduced lipolysis and PS bioaccessibility, while POPs bioaccessibility was increased thus suggesting a distinct preference for incorporation into mixed micelles. Elderly-specific modifications (gastric-intestinal phases) reduced POP bioaccessibility and lipolysis without affecting PS, which could be beneficial for the senior population’s health. Age-related differences in digestion processes are crucial when assessing nutritional impacts, emphasizing the complex interplay among enzymatic activity, food matrix, and physiological conditions. Further research is needed to refine dietary recommendations and enhance PS-enriched food’s efficacy across demographic groups.

## Abbreviations

CE: cholesterol esterase
DAG: diacylglycerols
E-STE: esterified sterols
FFA: free fatty acids
GL: gastric lipase
IS: internal standard
MAG: monoacylglycerols
POPs: plant sterols oxidation products
PS-WRB: plan sterols-enriched wholemeal rye bread
PS: plant sterols
SPE: solid-phase extraction
STE: free sterols
TAG: triacylglycerols
TOC: tocopherols
WRB: wholemeal rye bread
